# Mode of Delivery and Pregnancy Outcome in Women with Congenital Heart Disease

**DOI:** 10.1371/journal.pone.0167820

**Published:** 2016-12-22

**Authors:** Joris Hrycyk, Harald Kaemmerer, Nicole Nagdyman, Moritz Hamann, KTM Schneider, Bettina Kuschel

**Affiliations:** 1 Women’s Clinic (Frauenklinik), Klinikum Rechts der Isar, Technical University of Munich (Technische Universitaet Muenchen), Munich, Germany; 2 Department of Paediatric Cardiology and Congenital Heart Disease, German Heart Centre Munich (Deutsches Herzzentrum Muenchen), Technical University of Munich (Technische Universitaet Muenchen), Munich, Germany; Cincinnati Children's Hospital Medical Center, UNITED STATES

## Abstract

**Background:**

Advances in cardiac surgery and congenital cardiology have led to an increasing number of women with congenital heart disease (CHD) reaching childbearing age. In general, cardiologists recommend vaginal delivery for women with CHD to avoid complications from Caesarean section as many women with CHD tolerate vaginal delivery well.

**Methods and Results:**

This is a single-center study comparing mode of delivery, pregnancy outcome, indications for Caesarean section and induction of labor between women with and without CHD. A historical cohort study was conducted including 116 patients with CHD. An individual threefold matching with 348 women without CHD was carried out. Caesarean section was performed in 46.6% of pregnancies with CHD (33.6% without CHD, *P* = 0.012). Primary Caesarean section increases with severity of CHD (*P* = 0.036), 33.3% of women with CHD had primary planned Caesarean section due to cardiac reasons. Induction of labor was performed in 45.7% of attempted vaginal deliveries in women with CHD (27.9% without CHD, *P* = 0.001). Lower mean birth weight (*P* = 0.004) and Small for Gestational Age (SGA) (*P* < 0.001) were more common in women with CHD. One CHD patient suffered from postpartum hemorrhage.

**Conclusions:**

Concerns about maternal deterioration resulting in higher rates of induction of labor seem unjustified in most cases. Along with a possible reduction of Caesarean section on maternal request, a reduction of planned vaginal delivery may be expedient in reducing the rate of Caesarean section in women with CHD.

## Introduction

Advances in cardiology and cardiac surgery have led to a steadily increasing number of adults with congenital heart disease (CHD), including women, reaching childbearing age. Physiological hemodynamic changes during pregnancy and delivery are of special concern in this growing patient population. While many recent publications have recommended vaginal delivery as mode of choice for women with CHD [1–3], Caesarean section rate remains higher in these patients than in the normal population [3–5]. With Caesarean section rate among women with CHD varying widely between countries, Germany ranks above international average [3].

The objectives of the present single-center study were to compare mode of delivery, pregnancy outcome and indications for Caesarean section and induction of labor between women with and without CHD. Results from one of the large centers with regard to delivery in women with CHD may also help to ascertain the reasons for the comparatively high Caesarean section rate among women with CHD in Germany.

## Materials and Methods

A historical cohort study was conducted based on patient files, including 116 patients with CHD from the German Heart Centre Munich who delivered between January 2005 and December 2013 at the Klinikum rechts der Isar, Munich. An individual threefold matching with a total of 348 women without CHD was carried out, using the parameters maternal age, gravidity, parity, and year of delivery.

The study protocol was approved by the Ethics Committee of Klinikum rechts der Isar, Medizinische Fakultaet, Technical University of Munich (approval number 433/13).

CHD patients provided written consent to participate in the study.

Women with CHD were clinically classified by severity of CHD according to the American College of Cardiology [6] and functional class according to Perloff [7]. As for severity of CHD, primary cardiac main diagnosis (native CHD) was decisive for classification. As for functional class, the clinical cardiac status after surgical or interventional repair as evaluated last before delivery was decisive.

Indications for Caesarean sections were differentiated into cardiac, obstetric and other reasons. Cardiac reasons were defined as “poor heart function” and “worsening of heart function”, respectively. Obstetric reasons were defined as “fetal safety reasons” and, in the case of secondary Caesarean sections, “failure to progress in labor”. It should be noted, however, that this differentiation can be somewhat vague in clinical practice, given the great number of combined indications. The indication for Caesarean section as stated in the patient file by the obstetrician in charge was decisive.

Data analysis was performed using SPSS software version 22.0 (SPSS Inc., IBM). Chi square tests were used for comparing differences in categorical data between independent patient groups. Fisher’s exact tests were applied if any expected cell count was < 5. Student t tests were used for comparing differences in continuous data between independent patient groups. According to convention, *P* values < 0.05 were considered statistically significant.

## Results

### Characteristics of the study population

Maternal baseline characteristics in women with CHD are outlined in Table 1. Since maternal age and parity were among the matching parameters, mean maternal age and percentage of nulliparous and multiparous were the same in women without CHD.

**Table 1:** Maternal baseline characteristics in women with CHD.

		n = 116
		n (%)
Age (years) (SD)		29.7 (±4.5)
Parity		
	Nulliparous	85 (73.3)
	Multiparous	31 (26002E7)
Severity of CHD (according to ACC)		
	Simple	30 (25.9)
	Moderate	59 (50.9)
	Severe	27 (23.2)
Functional class (according to Perloff)		
	I	47 (40.5)
	II	66 (56.9)
	III	3 (2.6)
	IV	0 (0.0)
Diagnosis		
	Post-tricuspid shunts:	9 (7.8)
	VSD	
	Pre-tricuspid shunts:	17 (14.7)
	ASD, PFO	
	Left heart obstructions:	17 (14.7)
	AS, BAV, CoA, SAS	
	Right heart obstructions:	33 (28.4)
	DORV, PS, TOF	
	Complex anomalies:	24 (20.7)
	ccTGA, TGA, TOF/APV, TrA	
	Hereditary:	7 (6.0)
	Marfan’s syndrome	
	Congenital rhythm anomalies:	2 (1.7)
	atrioventricular block	
	Other:	7 (6.0)
	aortic aneurysm, Ebstein’s anomaly, congenital form of HOCM, MVP	

AS = aortic stenosis, ASD = atrial septal defect, BAV = bicuspid aortic valve, ccTGA = congenitally corrected transposition of the great arteries, CoA = coarctation of the aorta, DORV = double outlet right ventricle, HOCM = hypertrophic obstructive cardiomyopathy, MVP = mitral valve prolapse, PFO = patent foramen ovale, PS = pulmonary stenosis, SAS = subaortic stenosis, TGA = transposition of the great arteries, TOF = tetralogy of Fallot, TOF/APV = tetralogy of Fallot with absent pulmonary valve, TrA = truncus arteriosus, VSD = ventricular septal defect

Classification showed that severity of CHD and functional class did not correspond: With regard to severity, approximately half of the cases were considered “moderate“, with approximately a quarter of cases being considered “simple”and “severe”each [6]. With regard to the clinical cardiac status, 97.4% of cases belonged to functional classes I and II (with the bigger share in functional class II), and only 2.6% to functional class III, with no cases in a possible functional class IV [7].

Maternal risk factors for complications during and outcome of pregnancy are outlined in Table 2.

**Table 2:** Maternal risk factors.

		with CHD	without CHD	
		n = 116	n = 348	
		n (%)	n (%)	*P*
Body mass index (SD)		23,20 (±3,95)	22,76 (±3,86)	0.298
	≤17.5	0 (0.0)	9 (2.6)	0.120
	≥30.0	8 (6.9)	17 (4.9)	0.406
Smoking		1 (0.9)	6 (1.7)	0.686
Preeclampsia		1 (0.9)	11 (3.2)	0.310
Diabetes		2 (1.7)	23 (6.6)	0.044
	Preexisting	0 (0.0)	3 (0.9)	0.577
	Gestational	2 (1.7)	20 (5.7)	0.077
Prior C-section		11 (9.5)	17 (4.9)	0.072

Body mass index (BMI) was slightly higher in women with CHD. Smoking, preeclampsia and diabetes were more common in women without CHD, with a significant difference for diabetes only (*P* = 0.044). A total of 9.5% of patients with prior Caesarean section in women with CHD compared with 4.9% in women without CHD (*P* = 0.072).

### Mode of delivery

Details pertaining to mode of delivery are shown in Table 3. The rate of Caesarean section was significantly higher in women with CHD. Caesarean section was performed in 46.6% of pregnancies in women with CHD, compared with 33.6% of pregnancies in women without CHD (*P* = 0.012). The rate of primary Caesarean section, defined as “planned Caesarean section“, as well as the rate of secondary Caesarean section, defined as “Caesarean section following spontaneous onset of labor/failed attempted vaginal delivery”, was higher in women with CHD.

**Table 3:** Mode of delivery.

			with CHD	without CHD	
			n = 116	n = 348	
			n (%)	n (%)	*P*
Vaginal delivery			62 (53.4)	231 (66.4)	0.012
	Spontaneous		52 (44.8)	195 (56.0)	0.036
	Operative vaginal		10 (8.6)	36 (10.4)	0.590
		Vacuum extraction	10 (8.6)	33 (9.5)	0.782
		Forceps	0 (0.0)	3 (0.9)	0.577
C-section			54 (46.6)	117 (33.6)	0.012
	Primary		24 (20.7)	51 (14.7)	0.126
	Secondary		30 (25.9)	66 (18.9)	0.112

The rate of spontaneous vaginal delivery (44.8% vs. 56.0%, *P* = 0.036) as well as the rate of operative vaginal delivery (8.6% vs. 10.4%, *P* = 0.590) was lower in women with CHD.

Mode of delivery was compared in all multiparous patients according to prior Caesarean section (see Tables 4 and 5). In both women with and without CHD, Caesarean section rate was significantly higher in multiparous patients with prior Caesarean section compared with multiparous patients without prior Caesarean section (81.8% vs. 15.0% in women with CHD, *P* < 0.001, and 58.8% vs. 11.8% in women without CHD, *P* < 0.001, respectively).

**Table 4:** Mode of delivery according to prior Caesarean section in women with CHD.

		Prior C-section	No prior C-section	
		n = 11*	n = 20*	
		n (%)	n (%)	*P*
Vaginal delivery		2 (18.2)	17 (85.0)	< 0.001
C-section		9 (81.8)	3 (15.0)	< 0.001
	Primary	4 (36.4)	3 (15.0)	0.210
	Secondary	5 (45.5)	0 (0.0)	0.003

*Only patients with a possible prior Caesarean section, i.e. multiparous patients (n = 31 in patients with CHD), were included.

**Table 5:** Mode of delivery according to prior Caesarean section in women without CHD.

		Prior C-section	No prior C-section	
		n = 17*	n = 76*	
		n (%)	n (%)	*P*
Vaginal delivery		7 (41.2)	67 (88.2)	< 0.001
C-section		10 (58.8)	9 (11.8)	< 0.001
	Primary	4 (23.5)	2 (2.6)	0.010
	Secondary	6 (35.3)	7 (9.2)	0.012

*Only patients with a possible prior Caesarean section, i.e. multiparous patients (n = 93 in patients without CHD), were included.

In women with CHD, mode of delivery was compared according to division in groups for severity of CHD and functional class (see Tables 6 and 7). There was no correlation between severity of CHD and mode of delivery in general (*P* = 0.398). However, detailed analysis showed that the number of primary Caesarean sections increases significantly with severity (*P* = 0.036) while the number of secondary Caesarean sections decreases (*P* = 0.114).

**Table 6:** Mode of delivery according to severity of CHD.

		Severity of CHD			
		Simple	Moderate	Severe	
		n = 30	n = 59	n = 27	
		n (%)	n (%)	n (%)	*P*
Vaginal delivery		15 (50.0)	35 (59.3)	12 (44.4)	0.398
C-section		15 (50.0)	24 (40.7)	15 (55.6)	0.398
	Primary	3 (10.0)	11 (18.7)	10 (37.1)	0.036
	Secondary	12 (40.0)	13 (22.0)	5 (18.5)	0.114

**Table 7:** Mode of delivery according to heart function in women with CHD.

		Functional class			
		I	II	III	
		n = 47	n = 66	n = 3	
		n (%)	n (%)	n (%)	*P*
Vaginal delivery		30 (63.8)	31 (47.0)	1 (33.3)	0.146
C-section		17 (36.2)	35 (53.0)	2 (66.7)	0.146
	Primary	8 (17.0)	16 (24.2)	0 (0.0)	0.600
	Secondary	9 (19.2)	19 (28.8)	2 (66.7)	0.111

In the case of functional classes, the ratio of vaginal delivery and Caesarean section tended to invert according to worsening of the clinical cardiac status (*P* = 0.146).

Indications for primary and secondary Caesarean sections were examined individually (see Tables 8 and 9).

**Table 8:** Indications for primary Caesarean sections.

		with CHD	without CHD	
		n = 24	n = 51	
		n (%)	n (%)	*P*
Cardiac reason		8 (33.3)	-	< 0.001
Obstetric reason		9 (37.5)	43 (84.3)	< 0.001
Other reason		7 (29.2)	8 (15.7)	0.219
	Maternal request	4 (16.7)	5 (9.8)	0.455

**Table 9:** Indications for secondary Caesarean sections.

	with CHD	without CHD	
	n = 30	n = 66	
	n (%)	n (%)	*P*
Cardiac reason	2 (6.7)	-	0.095
Obstetric reason	27 (90.0)	62 (93.9)	0.674
Other reason	1 (3.3)	4 (6.1)	1.000

Cardiac reasons came to 33.3% of primary Caesarean sections in women with CHD. Obstetric reasons for primary Caesarean sections were significantly higher in women without CHD (*P* < 0.001). There was no significant difference in “other reasons” for primary Caesarean sections in women with and without CHD (*P* = 0.219). In both cases, Caesarean section on maternal request predominated with a relative percentage of 57.1% (n = 4 out of 7) and 62.5% (n = 5 out of 8), respectively.

In contrast to primary Caesarean sections, there was no significant difference in indications for secondary Caesarean sections in women with and without CHD. Cardiac reasons came to no more than 6.7% of secondary Caesarean sections in women with CHD. In women with and without CHD, obstetric reasons predominated clearly (90.0% vs. 93.9%).

### Induction of labor

Excluding primary Caesarean sections (20.7% vs. 14.7%), vaginal delivery was attempted in 79.3% of pregnancies in women with CHD and 85.3% of pregnancies in women without CHD. Details pertaining to induction of labor are shown in Table 10. Induction of labor was performed in 45.7% of attempted vaginal deliveries in women with CHD, compared with 27.9% in women without CHD (*P* = 0.001).

**Table 10:** Induction of labor.

			with CHD	without CHD	
			n = 92*	n = 297*	
			n (%)	n (%)	*P*
Induction of labor			42 (45.7)	83 (27.9)	0.001
Indication for induction of labor					
	Medical reason		27 (64.3)	82 (98.8)	< 0.001
	Logistical reason		15 (35.7)	1 (1.2)	< 0.001
		“because of long distance”	13 (31.0)	0 (0.0)	< 0.001

*To avoid falsification of results due to different rates of primary Caesarean sections in women with and without CHD (20.7% vs. 14.7%), only cases of attempted vaginal delivery (n = 389) were examined. 24 and 51 primary Caesarean sections, respectively, were excluded.

As can be seen in Tables 11 and 12, performance of induction of labor was associated with higher rates of secondary Caesarean section in both women with and without CHD. In women with CHD, secondary Caesarean section rate was 24.0% without induction of labor and 42.9% following induction (*P* = 0.055). In women without CHD, secondary Caesarean section rate was 17.8% without induction of labor and 33.7% following induction (*P* = 0.003).

**Table 11:** Mode of delivery according to performance of induction of labor in women with CHD.

	Induction performed	Induction not performed	
	n = 42*	n = 50*	
	n (%)	n (%)	*P*
Vaginal delivery	24 (57.1)	38 (76.0)	0.055
Secondary C-section	18 (42.9)	12 (24.0)	0.055

*As in Table 10, only cases of attempted vaginal delivery (n = 92 in patients with CHD) were examined.

**Table 12:** Mode of delivery according to performance of induction of labor in women without CHD.

	Induction performed	Induction not performed	
	n = 83*	n = 214*	
	n (%)	n (%)	P
Vaginal delivery	55 (66.3)	176 (82.2)	0.003
Secondary C-section	28 (33.7)	38 (17.8)	0.003

*As in Table 10, only cases of attempted vaginal delivery (n = 297 in patients without CHD) were examined.

Indications for induction of labor differed with high significance in women with and without CHD. In women without CHD, 98.8% of inductions of labor were performed for medical reasons, compared with only 64.3% in women with CHD (*P* < 0.001). Of the remaining 35.7% of inductions of labor which were performed for logistical reasons in women with CHD, a clear majority was performed “because of long distance”between the patient’s place of residence and our hospital, the relative percentage being 86.7% (n = 13 out of 15).

Mode of delivery following induction of labor was compared according to the different categories of severity of CHD and functional class, respectively (see Tables 13 and 14). As for severity of CHD, there was no correlation between mode of delivery following induction of labor (*P* = 0.843). In the case of functional classes, the ratio of vaginal delivery and secondary Caesarean sections tended to invert according to worsening of heart function (*P* = 0.254).

**Table 13:** Mode of delivery following induction of labor according to severity of CHD.

	Severity of CHD			
	Simple	Moderate	Severe	
	n = 14*	n = 21*	n =7*	
	n (%)	n (%)	n (%)	*P*
Vaginal delivery	7 (50.0)	13 (61.9)	4 (57.1)	0.843
Secondary C-section	7 (50.0)	8 (38.1)	3 (42.9)	0.843

*Total number of induced patients in each category

**Table 14:** Mode of delivery following induction of labor according to heart function in women with CHD.

	Functional class			
	I	II	III	
	n = 19*	n = 21*	n =2*	
	n (%)	n (%)	n (%)	*P*
Vaginal delivery	12 (63.2)	12 (57.1)	0 (0.0)	0.254
Secondary C-section	7 (36.8)	9 (42.9)	2 (100.0)	0.254

*Total number of induced patients in each category

### Pregnancy outcome

Pregnancy outcome is outlined in Table 15. There were no cases of maternal, perinatal or neonatal mortality, and no cases of adverse neonatal outcome in the present study. There was one case of adverse maternal outcome in the form of postpartum hemorrhage in a patient with CHD.

**Table 15:** Pregnancy outcome.

	with CHD	without CHD	
	n = 116	n = 348	
	n (%)	n (%)	*P*
Postpartum hemorrhage	1 (0.9)	0 (0.0)	0.250
Pregnancy duration (weeks) (SD)	38.7 (±2.3)	38.7 (±2.0)	0.054
Premature birth	13 (11.2)	32 (9.2)	0.526
Birth weight (g) (SD)	3092 (±588)	3267 (±547)	0.004
SGA	19 (16.4)	8 (2.3)	< 0.001
APGAR score (after 1 / 5 / 10 min)	8.32 / 9.21 / 9.65	8.25 / 9.18 / 9.65	
Arterial cord pH (SD)	7.28 (±0.08)	7.26 (±0.09)	0.017

Overall, mean pregnancy duration and the rate of premature birth were similar in women with and without CHD. Lower mean birth weight (*P* = 0.004) and Small for Gestational Age (SGA) (*P* < 0.001) were significantly more common in women with CHD.

## Discussion

Recommendation of vaginal delivery as mode of choice for women with CHD is based on different hemodynamic load during vaginal delivery and Caesarean section, resulting in significant cardiovascular changes—e.g. in Caesarean section under spinal anesthesia [8] and in more blood loss at delivery in Caesarean section [2]. Nevertheless, the present study shows a significantly higher rate of Caesarean section in women with CHD in comparison with women without CHD (46.6% vs. 33.6%, *P* = 0.012), which has already been described in previous population based studies, although to a lesser extent. Opotowsky et al. describe Caesarean section rates of 32.2% in women with CHD compared with 26.5% in women without CHD [4]. Thompson et al. describe 40.7% compared with 32.3% [5]. A possible explanation for the relatively high rate of Caesarean sections in women without CHD in the present study is that matching was carried out based on the sole characteristic of lacking CHD. Other risk factors more common in a university hospital’s patient collective were not taken into account separately. Against expectation, further analysis of common risk factors showed higher rates of diabetes (*P* = 0.044) and preeclampsia (*P* = 0.310) in the supposedly healthy cohort group. The impact of such risk factors as preeclampsia, HELLP syndrome, intrauterine growth restriction and other possible primary diseases can be comprehended by considering the high rate of “obstetric reasons” for primary Caesarean sections (84.3%) in women without CHD. Against expectation, further analysis showed a higher rate of obesity in women with CHD than in women without CHD (6.9% vs. 4.9%, *P* = 0.406). The rate of prior Caesarean section as well as the rate of Caesarean section after prior Caesarean section was higher in women with CHD, which in combination contributes to the higher overall Caesarean section rate in women with CHD.

Severity of CHD and heart function were analyzed as possible predictors for mode of delivery and pregnancy outcome.

The present study suggests that severity of CHD may be useful as a predictor for the risk of a primary Caesarean section. The rate of primary Caesarean sections increases with severity of CHD as could have been expected. It seems probable that women with a history of “severe” CHD will more likely be advised to consider an elective Caesarean section. In addition, due to their own experiences regarding hospitals and their medical past, they might preferably request a planned Caesarean section or a planned vaginal delivery by themselves.

Functional class on the other hand may be useful as a predictor for the risk of a secondary Caesarean section according to the present study. As could have been expected, poor clinical cardiac status seems more likely to lead to a failed attempted vaginal delivery. It could be shown that worsening of the clinical cardiac status correlates with (a) an increasing rate of Caesarean sections altogether, (b) an increasing rate of secondary Caesarean sections altogether as well as (c) an increasing rate of secondary Caesarean sections following induction of labor. Nevertheless, it has to be taken into account that these findings were not significant. The total number of cases in functional class III was very limited in the present study (n = 3), limiting the expressiveness of these findings.

Analysis of individual indications for primary and secondary Caesarean sections revealed “cardiac reasons” to be a significant factor for primary Caesarean sections only. With regard to secondary Caesarean sections, there was no difference between indications for women with and without CHD. These findings underline the assumption of severity of CHD being a predictor predominantly for primary Caesarean sections.

The present study shows higher rates of induction of labor in women with CHD as has been previously observed. Robertson et al. describe induction rates of 50% in women with heart disease compared with 28% in women without heart disease [1]. In general, the correlation between induction of labor and mode of delivery remains a matter under discussion with conflicting results [9]. In the largest systematic review and meta-analysis on the subject to date, including 37 randomized controlled trials, Wood et al. found lower Caesarean section rates following induction of labor. Nevertheless, the authors themselves emphasize the impact of non-treatment effects that might influence these results [9]. In studies that describe higher Caesarean section rates following induction of labor, multiple maternal influence factors like parity, maternal age and gestational age have been identified so far [10]. The present study shows higher Caesarean section rates following induction of labor, although not significantly. Analysis of individual indications for induction of labor, however, revealed a significant difference of indications between women with and without CHD. “Logistical reasons” were significantly higher in women with CHD, predominated by inductions “because of long distance” between residence and hospital, an indication completely missing in women without CHD.

Pregnancy outcome was similar in women with and without CHD in the present study. There were no adverse fetal outcomes, neither for Caesarean sections nor for vaginal deliveries. In the largest registry on the subject (Registry on Pregnancy and Cardiac Disease (ROPAC)) to date, analyzing data from 1321 pregnant women with structural heart disease, Ruys et al. found similar maternal outcomes and better fetal outcomes in planned vaginal delivery, thus recommending vaginal delivery for women with CHD [3]. Regarding the much smaller patient collective of the present study, no such difference could be observed. It should be kept in mind, however, that ROPAC included data from 60 hospitals in 28 countries, not all from very well developed countries and different levels of medical care.

The number of cases of SGA (19%) was similar to findings in previous studies [11, 12]. With regard to the impact of maternal cardiac function in fetal growth restriction, former studies had lower birth weight and more cases of SGA be expected in women with reduced maternal systolic function [13]. Higher rates of premature birth ranging from 17.5% [14] to 26.2% [11] were reported in previous studies. The present study could not find a significantly higher rate of premature birth in women with CHD than in women without CHD (11.2% vs. 9.2%, *P* = 0.526).

Apart from heart failure, postpartum hemorrhage had been identified as the major adverse maternal outcome in previous studies. Percentage ranged from 3% [14] to 8.4% [2]. The only case of postpartum hemorrhage in the present study occurred in a case of primary Caesarean section in a woman with CHD.

Generally speaking, congenital heart disease does not seem to have as big of an impact on delivery and pregnancy outcome as might have been expected. The same advances in medical care, particularly in cardiac surgery, that have led to the very growing patient population of pregnant women with CHD are also responsible for a high percentage of women with CHD showing good clinical cardiac status after treatment. Previous studies show equally small numbers of patients in functional classes III and IV. Siu et al. describe 4.0%, compared with 2.6% in the present study. This being said, patients in functional classes III and IV will most likely be considered desperately ill and be advised against pregnancy in the first place or be recommended having a termination in case of pregnancy, thus adding to this very outcome.

The analysis of indications for induction of labor showed a significantly higher rate of “planned vaginal delivery”, i.e. induction of labor for logistical reasons, in women with CHD. Future studies should delve into this finding more thoroughly, eventually by adding distance from hospital to the matching parameters. It remains to be investigated whether distance from hospital correlates with higher rates of induction of labor in all patients or in women with CHD only. As of now, the assumption could be made that induction of labor was more likely to be performed in women living far away from the hospital in presence of CHD out of concerns about maternal deterioration. Considering the aforementioned numbers of good heart function in women with CHD, such concerns resulting in higher rates of induction of labor [14] seem unjustified in most cases. A further investigation into whether distance from hospital is also a risk factor for secondary Caesarean section following induction of labor in all patients or in women with CHD only could corroborate the assumption that induction of labor seems more likely to be performed “prematurely” in women with CHD. In this case, a reduction of planned vaginal delivery—along with a possible reduction of Caesarean sections on maternal request and a possible higher rate of attempted vaginal delivery after prior Caesarean section—may be expedient in reducing the rate of Caesarean sections in women with CHD.

### Strengths and limitations

The present study’s main strength is that women with CHD were matched with a large patient collective of women without CHD who delivered during the same period of time at the same center. That way, disturbing factors like differences or changes in obstetrical management could be eliminated.

A limitation of the present study is that it was conducted as a historical cohort study. Data analysis was performed retrospectively based on patient files with the possibility of incorrect data. The conclusions of the present study are limited by the fact that study participants were recruited from a specialized tertiary care center for adults with CHD. Presumably, this results in more cases of complex CHD than in the general patient population of women with CHD seen by cardiologists in private practice and other centers. As shown above, more cases of “severe” CHD may also be responsible for more cases of planned Caesarean sections as well as planned vaginal delivery. As in every single-center study, patient numbers were limited, especially those of women with CHD belonging to functional classes III and IV. Although the majority of patients belonging to functional classes III and IV will be advised against pregnancy due to high morbidity and/or mortality risks in the first place, future studies should involve greater numbers of patients, prospectively collected from multiple centers.
